# C-reactive protein or procalcitonin combined with rhinorrhea for discrimination of viral from bacterial infections in hospitalized adults in non-intensive care units with lower respiratory tract infections

**DOI:** 10.1186/s12890-021-01672-7

**Published:** 2021-09-28

**Authors:** Shengchen Duan, Xiaoying Gu, Guohui Fan, Fei Zhou, Guangfa Zhu, Bin Cao

**Affiliations:** 1 Department of Pulmonary and Critical Care Medicine, Beijing Anzhen Hospital, Capital Medical University, Beijing, China; 2 Department of Institute of Clinical Medical Sciences, China-Japan Friendship Hospital, Beijing, China; 3 Institute of Respiratory Medicine, Chinese Academy of Medical Science, Beijing, China; 4 National Clinical Research Center of Respiratory Diseases, Beijing, China; 5 Department of Pulmonary and Critical Care Medicine, China-Japan Friendship Hospital, Beijing, China; 6 Laboratory of Clinical Microbiology and Infectious Diseases, China-Japan Friendship Hospital, Beijing, China; 7 Clinical Center for Pulmonary Infections, Capital Medical University, Beijing, China; 8 Tsinghua University-Peking University Joint Center for Life Sciences, East Yinghua Road, Chaoyang District, Beijing, 100029 China

**Keywords:** C-reactive protein, Procalcitonin, Clinical characteristics, Rhinorrhea, Lower respiratory tract infection

## Abstract

**Background:**

Whether procalcitonin (PCT) or C-reactive protein (CRP) combined with certain clinical characteristics can better distinguish viral from bacterial infections remains unclear. The aim of the study was to assess the ability of PCT or CRP combined with clinical characteristics to distinguish between viral and bacterial infections in hospitalized non-intensive care unit (ICU) adults with lower respiratory tract infection (LRTI).

**Methods:**

This was a post-hoc analysis of a randomized clinical trial previously conducted among LRTI patients. The ability of PCT, CRP and PCT or CRP combined with clinical symptoms to discriminate between viral and bacterial infection were assessed by portraying receiver operating characteristic (ROC) curves among patients with only a viral or a typical bacterial infection.

**Results:**

In total, 209 infected patients (viral 69%, bacterial 31%) were included in the study. When using CRP or PCT to discriminate between viral and bacterial LRTI, the optimal cut-off points were 22 mg/L and 0.18 ng/mL, respectively. When the optimal cut-off for CRP (≤ 22 mg/L) or PCT (≤ 0.18 ng/mL) combined with rhinorrhea was used to discriminate viral from bacterial LRTI, the AUCs were 0.81 (95% CI: 0.75–0.87) and 0.80 (95% CI: 0.74–0.86), which was statistically significantly better than when CRP or PCT used alone (*p* < 0.001). When CRP ≤ 22 mg/L, PCT ≤ 0.18 ng/mL and rhinorrhea were combined, the AUC was 0.86 (95% CI: 0.80–0.91), which was statistically significantly higher than when CRP (≤ 22 mg/L) or PCT (≤ 0.18 ng/mL) was combined with rhinorrhea (*p* = 0.011 and *p* = 0.021).

**Conclusions:**

Either CRP ≤ 22 mg/L or PCT ≤ 0.18 ng/mL combined with rhinorrhea could help distinguish viral from bacterial infections in hospitalized non-ICU adults with LRTI. When rhinorrhea was combined together, discrimination ability was further improved.

## Background

Lower respiratory tract infection (LRTI) is the most common infectious disease that may cause death, accounting for about three million deaths worldwide in 2020 [1]. Viral infection is one of the most important causes of LRTI. Identifying the pathogens involved in a timely manner is essential for selecting antibiotics, as a detection delay may potentially result in antimicrobial resistance. Antimicrobial resistance can cause a corresponding financial burden and environmental pollution, especially when antibiotics are inappropriately prescribed to patients with viral infections [2].

Although some novel molecular diagnostic or culture-independent assays offer enhanced opportunities to identify respiratory pathogens, researchers are still pursuing much simpler, faster and cheaper ways to identify different pathogens. Serum markers such as C-reactive protein (CRP) and procalcitonin (PCT), which can help guide antibiotic use in LRTI patients, have been studied most often [3, 4]. But whether PCT or CRP could distinguish viral or bacterial infection is a controversial issue [5–8]. Furthermore, most of the studies have been hampered by an incomplete etiologic approach, because only a limited number of infectious agents have been assessed or techniques with low sensitivity have been used [9, 10]. Consequently, those studies have not reported reliable information on the use of biomarkers for differentiating bacterial from viral LRTIs.

Though some overlaps exist in symptoms and clinical presentation between bacterial and viral infection, viral infections produce characteristic symptoms, such as headache, generalized muscle pain and rhinorrhea. One recent study reported that a combination of clinical symptoms and blood biomarkers can distinguish bacterial from viral community-acquired pneumonia in children [11]. However, to date few studies have been conducted for adult LRTIs.

The aim of the present study was to assess whether PCT or CRP combined with clinical characteristics could distinguish between viral and bacterial infections using comprehensive and sensitive methods of etiologic classification in hospitalized non-intensive care unit (ICU) adults with LRTIs.

## Methods

### Study design

This was a post hoc analysis of a randomized controlled trial (RCT) that had been previously published [12]. The RCT took place between October 2017 and July 2018 in the China-Japan Friendship Hospital (CJFH), Beijing, China (clinicaltrials.gov identifier: NCT03391076). The RCT was approved by the ethics committee of CJFH (2017-29). Written informed consent was obtained from each patient after they had met the inclusion criteria.

### RCT population

The inclusion criteria of the RCT study were as follows: hospitalized patients aged ≥ 18 years who were preliminarily diagnosed with radiographically confirmed community acquired pneumonia (CAP); acute exacerbation of chronic obstructive pulmonary disease (AECOPD); or acute exacerbation of bronchiectasis, recruited on the day of hospitalization. Patients were excluded if they were: < 18 years old; pregnant; had hospital acquired pneumonia; or lung tuberculosis. We also excluded immunosuppressed patients. In addition, patients with any other condition that may have increased serum PCT levels were also excluded. For this post hoc analysis, patients who did at least one bacterial and one viral test were recruited. In addition, patients without CRP or PCT test results, or not having bacterial or viral pathogens detected were further excluded.

### PCT and CRP measurements

PCT or CRP concentrations were measured in the clinical laboratory of CJFH within 24 h of admission. CRP was measured using a high-sensitive-CRP Kit (i-Reader, China). The upper and lower detection limits were 200 mg/L and 1 mg/L, respectively. PCT was measured using a PCT Kit (i-Reader, China), with a detection limit of 0.01 ng/mL.

### Pathogen testing

Bacterial and fungal tests included: culture of qualified sputum specimens; lower respiratory tract specimens; pleural fluid samples or blood; *Streptococcus* detected by urinary antigen (BinaxNOW, Alere). Atypical bacteria testing for *Mycoplasma pneumoniae* (MP) and *Chlamydophila pneumoniae* (CP) included the reverse transcriptase polymerase chain reaction (RT-PCR) of sputum or other lower respiratory tract specimens, and nasopharyngeal swabs using FilmArray respiratory panel (FARP) and urinary antigen for *Legionella pneumophila* (BinaxNOW, Alere). Mycobacterial tests included acid-fast bacillus culture and mycobacteria nucleic acid detection (Xpert MTB/RIF).

Viral tests included FARP) of nasopharyngeal swabs for influenza A (H1 and H3) virus, influenza B virus, respiratory syncytial virus, rhinovirus or enterovirus, human metapneumovirus, parainfluenza virus types 1–4, coronaviruses (OC43, 229E, HKU1, and NL63), and adenovirus; RT-PCR of sputum, oropharyngeal/nasopharyngeal swabs, or other lower respiratory tract specimen samples for influenza A (H1N1, H7N9) virus, influenza B virus, respiratory syncytial virus, parainfluenza virus, adenovirus, Epstein-Barr virus, herpes simplex virus, and human cytomegalovirus; rapid antigen assay of influenza virus (BinaxNOW, Alere) in oropharyngeal/ nasopharyngeal swabs, or qualified lower respiratory tract specimens.

### Statistical analysis

Patients were divided into two groups according to the pathogen detection results. Those patients with bacteria detected and negative mycobacterial/fungal tests results, regardless of viral or atypical bacteria results, were classified into the bacteria group. The other patients only with viruses detected were classified into the virus group.

Baseline characteristics were expressed as a number (proportion) or median (interquartile range) and compared by χ2 or the Mann–Whitney U test when appropriate. We then assessed the predictive performance of CRP, PCT and PCT combined with CRP for discriminating viral from bacterial infection by plotting receiver operating characteristic (ROC) curves. Optimal cut-off points for CRP and PCT were defined as the point on the ROC curve that had the maximum Youden index. Furthermore, according to the optimal cut-off points, the performance of PCT ≤ 0.18 ng/L, CRP ≤ 22 mg/L, PCT ≤ 0.18 ng/L and CRP ≤ 22 mg/L combined with significant clinical features to discriminate viral and bacterial infections were evaluated. Sensitivity, specificity, positive predictive value (PPV), negative predictive value (NPV) and their 95% confidence intervals (CIs) were calculated. The areas under the curve (AUCs) and 95% CIs were estimated and compared to determine the different discrimination models. A two-sided α less than 0.05 was considered to be statistically significant for all statistical tests. Statistical analyses were performed using SAS software, version 9.4 (SAS Institute Inc.), unless otherwise indicated.

## Results

**Fig. 1:**
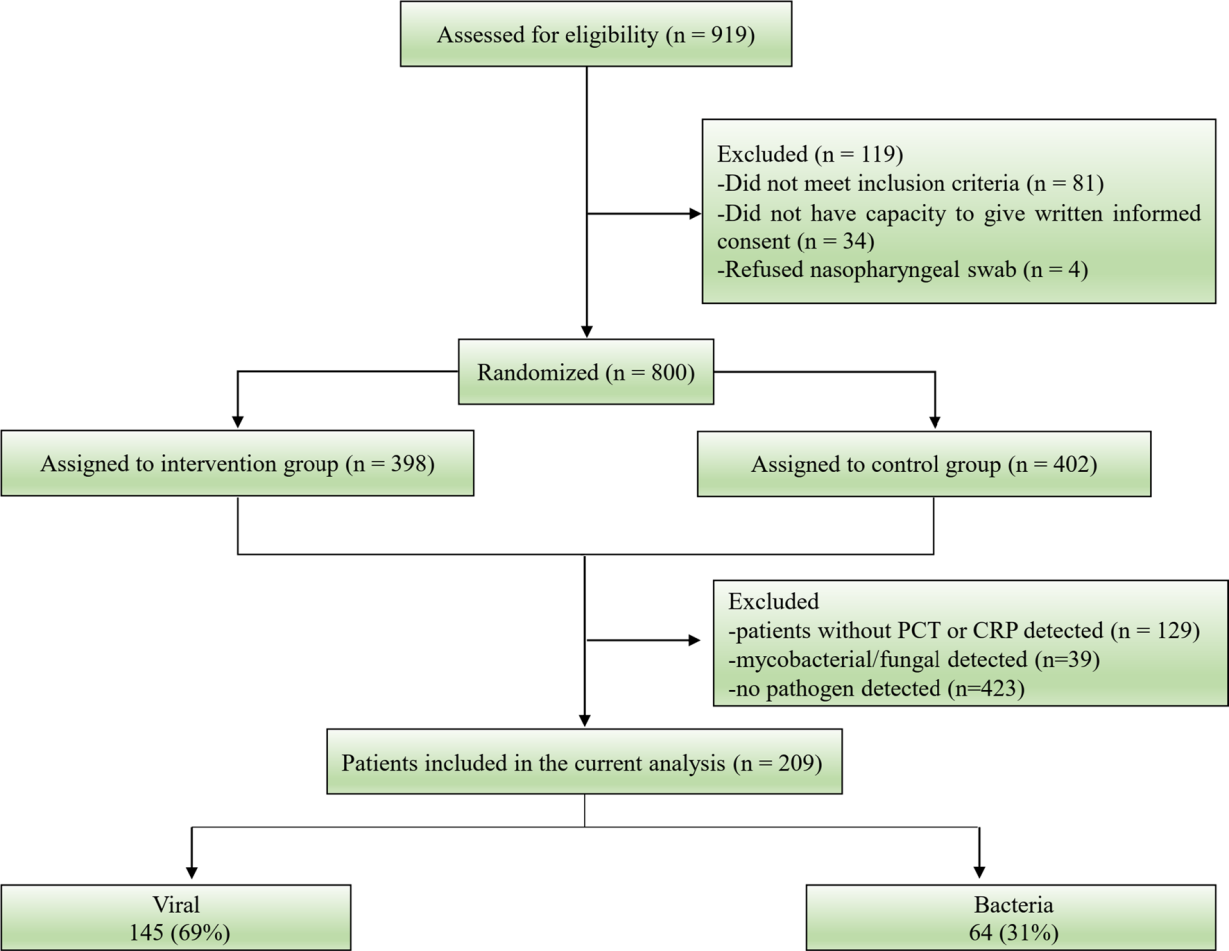
Flow diagram of patients included in the study

Between Oct 16, 2017 and Jul 13, 2018, we recruited 800 patients from the previous RCT study. After excluding 129 patients without PCT or CRP, 39 patients with mycobacterial/fungal infections were detected, 423 patients with no pathogens detected, with 209 patients included in the current analysis. Of these patients, the viral group accounted for 69% and the bacteria group 31% (Fig. 1).

The baseline characteristics of patients in the viral and bacterial groups are shown in Table 1. The proportions of patients with headache or rhinorrhea was higher in patients infected with a virus than that patients infected with bacteria (36.6%, 53/145 vs. 18.8%, 12/64; *p* = 0.010 and 55.2%, 80/145 vs. 20.3%, 13/64; *p* < 0.000). The proportions of CAP patients in the virus and bacteria groups were 60.0% (87/145) and 35.9% (23/64), respectively. The corresponding proportions of AECOPD patients were 26.2% (38/145) and 32.8% (21/64), and 13.7% (20/145) and 31.3% (20/64) for acute exacerbation of bronchiectasis patients, respectively. Both median PCT and CRP were significantly lower in the virus group than in the bacteria group (0.1 ng/mL (0.1, 0.2) vs. 0.3 ng/mL (0.2, 0.7); *p* < 0.000 and 10.4 mg/L (4.0, 28.0) vs. 53.0 mg/L (23.0, 96.7); *p* = 0.000). And median neutrophil count in the virus group was lower than that in the bacteria group (4.4 × 10^9^/L (2.7, 6.7) vs. 4.9 × 10^9^/L (4.0, 7.3); *p* = 0.009).

**Table 1:** Demographic and clinical characteristics

Variable	Virus (N = 145)	Bacteria (N = 64)	*p* value
Age (years)	64 (48, 78)	64(54, 76)	0.8319
Male (%)	78 (54)	42 (66)	0.1820
**Observation**			
BMI (kg/cm^2^)	23.4 (20.4, 25.9)	22.2 (18.6, 26.0)	0.3168
Respiratory frequency (bpm)	20 (20, 22)	20 (20, 21)	0.6199
Heart rate (bpm)	90 (80, 100)	95 (80, 102)	0.2724
Fever (%)	105 (72.4)	39 (60.9)	0.0985
Cough (%)	142 (97.9)	63 (98.4)	0.8018
Chest pain (%)	36 (24.8)	15 (23.4)	0.8293
Headache (%)	53 (36.6)	12 (18.8)	0.0104
Rhinorrhea (%)	80 (55.2)	13 (20.3)	< .0001
Final diagnosis (%)			< .0001
CAP	87/145 (60.0)	23/64 (35.9)	
AECOPD	38/145 (26.2)	21/64 (32.8)	
AE of bronchiectasis	20/145 (13.7)	20/64 (31.3)	
Dyspnea (%)	110 (75.9)	53 (82.8)	0.2636
Diarrhea (%)	25 (17.2)	6 (9.4)	0.1403
**Comorbidity (%)**			
Cardiovascular disease	66 (45.5)	32 (50.0)	0.5495
Diabetes	32 (22.1)	19 (29.7)	0.2372
Renal disease	9 (6.2)	3 (4.7)	0.6577
Liver disease	2 (1.4)	1 (1.6)	0.9189
Cancer	9 (6.2)	4 (6.3)	0.2493
Current smoker	24 (16.6)	10 (15.6)	0.8671
Influenza vaccine(< 1 year)	14 (9.7)	11 (17.2)	0.1219
**Laboratory test**			
White blood cell count(*10^9^/L)	6.8 (4.8, 9.5)	7.2 (5.8, 9.7)	0.0885
Lymphocyte count(*10^9^/L)	1.2 (0.8, 1.7)	1.3 (0.9, 1.8)	0.1523
Neutrophil count(*10^9^/L)	4.4 (2.7, 6.7)	4.9 (4.0, 7.3)	0.0087
Procalcitonin(ng/mL)	0.1 (0.1,0.2)	0.3 (0.2,0.7)	< .0001
C-reactive protein(mg/L)	10.4 (4.0,28.0)	53.0 (23.0,96.7)	0.0001

Data are presented as median (interquartile range) for continuous variables and as percent for categorical variables

Categorical variables were compared using χ^2^ tests, and continuous variables were compared using Wilcoxon rank-sum test or Student’s *t* test

*AE* acute exacerbation; *AECOPD* acute exacerbation of chronic obstructive pulmonary disease; *CAP* community-acquired pneumonia

**Fig. 2:**
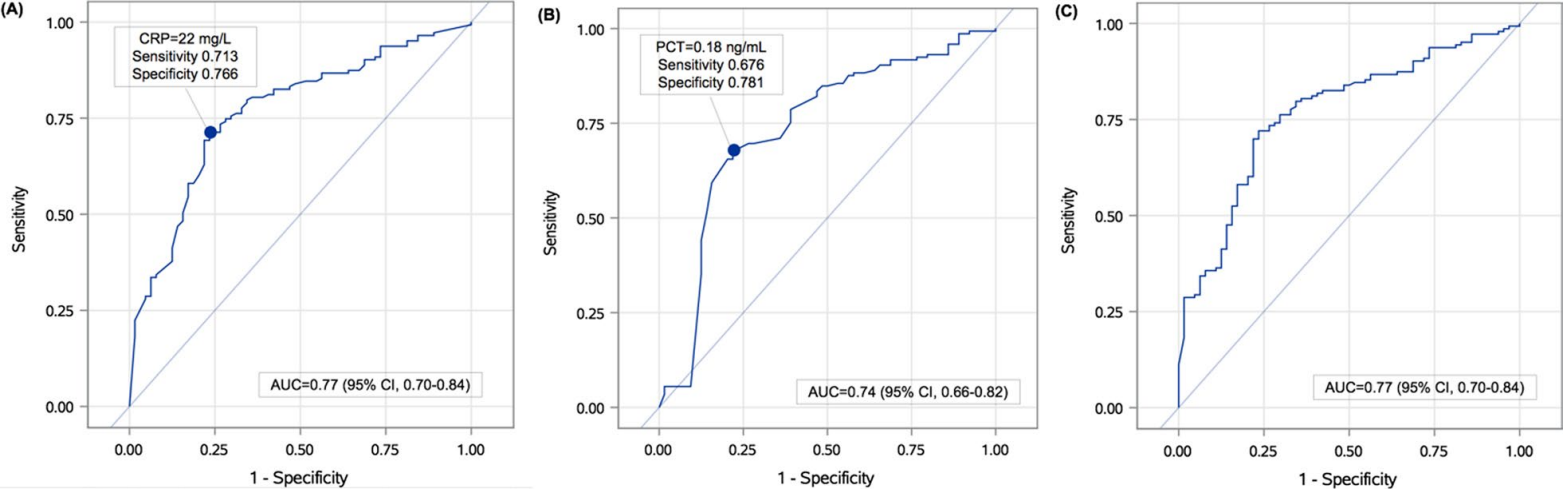
Comparison of receiver operating characteristic curves for CRP (**A**), PCT (**B**), and CRP combined with PCT to discriminate viral from bacterial lower respiratory tract infection (**C**)

When using CRP to discriminate viral from bacteria LRTI, the area under the ROC curve was 0.77 (95% CI: 0.70–0.84), and the optimal CRP cut-off point was 22 mg/L (Fig. 2A). Regarding PCT, the area under the ROC curve was 0.74 (95% CI: 0.66–0.82), and the optimal PCT cut-off point was 0.18 ng/mL (Fig. 2B). When CRP (≤ 22 mg/L) was combined with PCT (≤ 0.18 ng/mL) to discriminate viral from bacteria LRTIs, the area under the ROC curve was 0.77 (95% CI: 0.70–0.84) (Fig. 2C).

We used the optimal cut-off for CRP or PCT combined with headache or rhinorrhea to discriminate viral from bacterial LRTIs (Table 2). The areas under the ROC curve were 0.81 (95% CI: 0.75–0.87) and 0.80 (95% CI: 0.74–0.86), respectively when CRP (≤ 22 mg/L) or PCT (≤ 0.18 ng/mL) was combined with rhinorrhea, which was statistically significantly better than when CRP or PCT alone (*p* < 0.001). When CRP (≤ 22 mg/L), PCT (≤ 0.18 ng/mL) and rhinorrhea were combined together, the area under the ROC curve was 0.86 (95% CI: 0.80–0.91), which were statistically significantly greater than when CRP (≤ 22 mg/L) or PCT (≤ 0.18 ng/mL) was combined with rhinorrhea to distinguish between viral and bacterial LRTIs (*p* = 0.011 and *p* = 0.021).

**Table 2:** Sensitivity, specificity, positive predictive value (PPV) and negative predictive value (NPV) for optimal cut-off values of CRP and PCT level to differentiate viral from bacterial LRTI

	CRP cut-off level			PCT cut-off level			CRP and PCT cut-off level
	≤ 22 mg/L alone	≤ 22 mg/L and Headache	≤ 22 mg/L and Rhinorrhea	≤ 0.18 ng/mL alone	≤ 0.18 ng/mL and Headache	≤ 0.18 ng/mL and Rhinorrhea	CRP ≤ 22 mg/L combined PCT ≤ 0.18 ng/mL and Rhinorrhea
Sensitivity	0.71 (0.64–0.78)	0.71 (0.64–0.78)	0.90 (0.85–0.95)	0.68 (0.60–0.76)	0.68 (0.60–0.76)	0.86 (0.80–0.92)	0.73 (0.66–0.80)
Specificity	0.77 (0.67–0.87)	0.77 (0.67–0.87)	0.63 (0.51–0.75)	0.78 (0.68–0.88)	0.78 (0.68–0.88)	0.61 (0.49–0.73)	0.88 (0.80–0.96)
PPV	0.87 (0.81–0.93)	0.87 (0.81–0.93)	0.84 (0.78–0.90)	0.88 (0.81–0.94)	0.88 (0.81–0.94)	0.83 (0.77–0.89)	0.93 (0.88–0.98)
NPV	0.54 (0.44–0.65)	0.54 (0.44–0.65)	0.73 (0.61–0.84)	0.52 (0.42–0.61)	0.52 (0.42–0.61)	0.65 (0.53–0.77)	0.59 (0.49–0.69)
AUC	0.74 (0.68–0.80)*	0.78 (0.71–0.84)	0.81 (0.75–0.87)**	0.73 (0.67–0.79)^#^	0.75 (0.69–0.82)	0.80 (0.74–0.86)^##^	0.86 (0.80–0.91)^&^

^**^versus *p < 0.0001

^##^versus ^#^*p* < 0.0001

^**^versus ^&^*p* = 0.0107

^##^versus ^&^*p* = 0.020

## Discussion

With the etiological detection approach covering a relatively wide spectrum of pathogens in the study, it was found that either CRP ≤ 22 mg/L or PCT ≤ 0.18 ng/mL combined with rhinorrhea could discriminate viral from bacterial infection in hospitalized non-ICU adults with LRTIs, a topic which has rarely been explored in adults. When a CRP concentration ≤ 22 mg/L, PCT ≤ 0.18 ng/mL and rhinorrhea are considered together, discrimination of viral from bacterial infection was further improved.

For many years, physicians have sought to find a marker that could help discriminate virus from bacterial infection. CRP is a homopentameric acute-phase inflammatory protein, which reacts with the capsular (C)-polysaccharide of *Pneumococcus.* In the presence of calcium, CRP binds to polysaccharides such as phosphocholine on microorganisms and triggers the classical complement pathway of innate immunity by activating C1q and can respond quickly to bacterial infection [13]. Therefore, CRP was considered to be able to distinguish between viral and bacterial infections in the 1990s [14, 15]. But with the relative progress of detection technology, further studies indicated that CRP could not distinguish a viral infection from a bacterial infection [11, 16, 17]. Having reviewed these studies, most of them were conducted in pediatric patients, and the pathogen detection test had a low sensitivity and covered limited types of pathogens. In the present study of adults hospitalized with LRTIs, we used RT-PCR, multiple nested PCR and FARP testing, because they are highly sensitive and accurate methods to detect viruses and atypical bacteria. Furthermore, the types of pathogens we detected were very comprehensive. Based on the findings, we suggest that our grouping was more accurate and the results more credible than those of previous studies. We found the optimal CRP cut-off point was 22 mg/L, but that it alone could not identify whether it was a viral or bacterial infection in our patients.

During normal homeostasis, pre-procalcitonin undergoes initial synthesis in thyroid C cells. Later this peptide is transformed into procalcitonin after cleavage of a 25-amino acid signal sequence by endopeptidases. Normally, physiological conditions results in very low serum procalcitonin levels (< 0.05 ng/mL). However, the synthesis of PCT can be increased (100- to 1,000-fold) as a result of endotoxins and/or cytokines (e.g., Interleukin-6, tumor necrosis factor-α), which have multiple actions on various tissues. The extra-thyroid synthesis of PCT has been found to occur in the liver, pancreas, kidney, lung, intestine and within leukocytes; however, it is noteworthy that the synthesis of PCT is suppressed in these tissues in the absence of bacterial infection. In contrast, cytokines, such as interferon-gamma, which are secreted during a viral infection, lead to down-regulation of PCT assays [18, 19]. Combined with the above mechanism, PCT is a widely used and recognized biomarker of bacterial infection. Though PCT can guide antibiotic use to treat respiratory tract infections, therapy that has been widely adopted throughout the world [18], a number of recently published studies reported that PCT could not distinguish between viral and bacterial infections [8, 20]. The study of Self, which used sensitive and widely available etiological detection methods, found no procalcitonin threshold that could perfectly discriminate between viral and bacterial pathogens [8]. A meta-analysis found that the sensitivity and specificity of PCT were 0.55 and 0.76 in distinguishing viruses from bacteria in CAP patients. However, it is not sufficient reliable evidence either to mandate administration of antibiotics or to enable withholding such treatment in patients with CAP [20]. Our results revealed that the optimal PCT cut-off point was 0.18 ng/mL, but it may not be an ideal marker to distinguish viral from bacterial infections. This viewpoint is consistent with the conclusion of the study by Self. Therefore, we believe the use of PCT alone to identify a bacterial or viral infections and to guide the use of antibiotics selection should be treated with caution.

With increased interest in PCT research, many studies have shown that CRP is inferior to PCT in identifying bacterial or viral infections [6, 17, 21]. In our study, we found that CRP is non-inferior to PCT in differentiating viral from bacterial infection in LRTI patients. Recently, one RCT found that CRP-guided prescribing of antibiotics for AECOPD resulted in a lower percentage of patients with no evidence of harm [4]. Another study showed that the provision of PCT assay results in addition to usual care did not result in lower use of antibiotics than usual care in patients with suspected LRTIs [22]. Combined with our results, there is a clear need to examine further the importance of CRP in identifying viral infections and guiding antibiotic use, in part because it is readily available and cheaper than PCT.

The most important finding of the present study was that a concentration of CRP ≤ 22 mg/L or PCT ≤ 0.18 ng/mL combined with rhinorrhea could help to discriminate a bacterial infection from a viral infection. A study of children found that compared to CRP concentration ≥ 72 mg/L alone, CRP ≥ 72 mg/L combined with symptoms (including rhinorrhea) could improve the specificity and PPV in discriminating bacterial from viral pneumonia [11]. Some factors indicate why the CRP optimal cut-off point in our study was lower than that proposed by Bhuiyan [11]. First, the types of patients and diseases were different in the two studies. Second, the proportion of patients who received antibiotic therapy was high before hospitalization and the onset of illness to hospital admission was long (7 days) in our study, which may have influenced the CRP value [12]. Though antiviral drugs and virus detection methods have limited use in the clinic at present, clinicians should be encouraged to believe that if a LRTI patient has a low CRP, or PCT combined with rhinorrhea, to have more confidence in stopping or changing antimicrobial therapy.

Our study has a number of limitations. First, it is a reanalysis of a previous RCT, and not all enrolled patients received the FARP test. Second, a large proportion of patients, who had no pathogen detected, were excluded from the current analysis although we did an etiology-based study. Third, the study was conducted in general wards, without including patients from ICUs. In view of these limitations, extrapolation of our results should be carefully interpreted. Further extensive research will be necessary in the future to verify the accuracy of our conclusions.

## Conclusions

Either CRP ≤ 22 mg/L or PCT ≤ 0.18 ng/mL combined with rhinorrhea helps to distinguish a viral infection from a bacterial infection in hospitalized non-ICU adults with LRTIs. When the CRP concentration was ≤ 22 mg/L, PCT ≤ 0.18 ng/mL and rhinorrhea data combined together, discrimination of a viral infection from a bacterial infection was further improved.

## Data Availability

As other investigations involving this data are in progress, the data will not be available to others at present. When all investigations are finished, the data will be made available on reasonable request.

## Abbreviations

CRP: C-reactive protein
PCT: Procalcitonin
LRTI: Lower respiratory tract infection
ICU: Intensive care unit
ROC: Receiver operating characteristic
RCT: Randomized controlled trial
CJFH: China-Japan Friendship Hospital
CAP: Community acquired pneumonia
AECOPD: Acute exacerbation of chronic obstructive pulmonary disease
MP: Mycoplasma pneumonia
CP: Chlamydophila pneumonia
RT-PCR: Reverse transcriptase polymerase chain reaction
PPV: Positive predictive value
NPV: Negative predictive value
CIs: Confidence intervals
AUCs: Areas under the curve
